# Malignant Solitary Fibrous Tumor of the Kidney: Report of the First Case Managed with Interferon

**DOI:** 10.1155/2013/564980

**Published:** 2013-01-15

**Authors:** Javier Cuello, Ricardo Brugés

**Affiliations:** ^1^Clinical Oncology Group, Cancerology National Institute, E.S.E., Bogota, Colombia; ^2^El Bosque University, Bogota, Colombia

## Abstract

Solitary fibrous tumors of the kidney are extremely rare tumors with unpredictable behavior. We describe a case of a patient with a solitary fibrous tumor of kidney with malignant findings with distant metastasis and nephrectomy managed with subcutaneous interferon achieving 23 months of progression-free survival. To date there is no prospective evaluation of any specific modality of treatment, but the surgical management and long-term followup are the only ones so far recommended strategies in the management of these patients. Studies are awaited with more patients to evaluate the different strategies of systemic therapy reported so far to allow adding survival benefit.

## 1. Introduction

Solitary fibrous tumors are rare mesenchymal neoplasms, considered a variant of hemangiopericytomas usually originates in the pleura; however, there have been reports of extrapleural origin (abdomen, retroperitoneum, upper extremities, orbit, cervix, meninges, mediastinum, parotid, nasal cavity, neck, etc.) [1–4]. The location is even more rare urogenital, and according to the literature, only 49 cases of solitary fibrous tumor of the kidney have been reported in the literature [5]. The origin of the majority of cases formed in the kidney is found in the renal capsule tissue or connective tissue interstitial peripelvis. Most cases presented with suspected renal cell neoplasms; however, morphologically, solitary fibrous tumors are characterized by the proliferation of spindle cells with little pattern in their architecture, and the final diagnosis was made with immunohistochemical findings that show staining for CD34 [6–9].

## 2. Case Report

**Figure 1:**
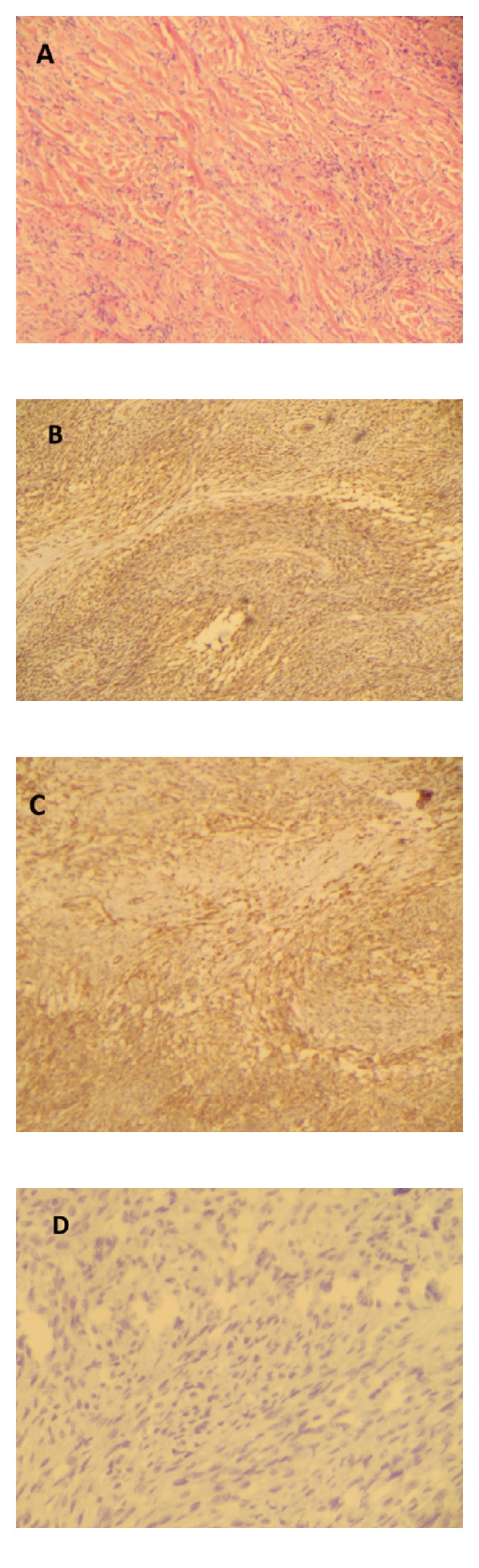
Immunohistochemical evidence CD34 positive cells (panel B), vimentin (panel C), and negative for S100 (panel D).

A 49 year old woman with no history of importance who enters the emergency room by 2-months of dyspnea at rest, associated with pleuritic chest pain on right chest, dry cough, no fever. The chest radiograph showed a right pleural effusion and multiple nodular lesions on the pleura, so we decided to perform a CT chest and abdomen in which evidenced free right pleural effusion occupying 80% of the right chest, at least two pleural masses with solid density that capture the contrast, multiple pulmonary nodules with soft tissue density in both lungs and left kidney mass. Carried left nephrectomy with suspected metastatic renal carcinoma, shows renal mass plus liver metastases which were resected. The analysis of the tumor presented as a first option versus solitary fibrous tumor angiomyolipoma. Immunohistochemical studies that showed cell reactivity with CD34, CD99, BCL-2, and vimentin are negative for HMB-45, AMS, CD68, cytokeratin cocktail, and S100, and the Ki67 is not assessable (Figure 1). This profile supports the histological diagnosis of solitary fibrous tumor with origin in the kidney.

We decided to start treatment with interferon a2b subcutaneous dose, extrapolating the cases of patients with solitary fibrous tumor of the pleura [12]. The dose was adjusted for flu symptoms and she is with stable disease at 23 months follow-up.

## 3. Discussion

Solitary fibrous tumors are extremely rare tumors, arising mostly at the level of the pleura, and cases arising from the urogenital region are even more rare, with 49 cases reported so far in the literature. The histogenesis of this entity is still unknown, but recent studies suggest a primitive mesenchymal cells or level perivascular [10, 11].

The differential diagnosis of these cases includes sarcomatoid variant of renal carcinoma, angiomyolipoma, fibromas, and fibrosarcomas.  Table 1 presents the cases so far published, reporting the primary source, and histologic variant outcomes.

These results show a relatively rare entity, with peak presentation in the fifth decade of life, arising mostly in the renal parenchyma unilaterally (Table 2).

About 14% of patients have aggressive behavior where common management strategy is nephrectomy with complete resection of the lesions. The pathological findings that have been correlated with aggressive behavior are pleomorphism, increased cellularity and mitotic activity (>4 mitosis/10 high-power fields), necrosis, hemorrhage, and atypical sites (parietal pleura, lung parenchyma) [13]. However, even the clinical behavior can not accurately predict the histopathological findings, as some cases with results suggesting benign disease may show aggressive behavior and vice versa, so it is necessary that these patients have an indefinite period of observation [14–19].

In relation to the management of this condition, there is as yet no prospective evaluation of any specific treatment modality; however, case reports and retrospective case series suggest that complete surgical resection and long-term followup are generally most recommended strategies. In cases such as the present, which present with metastatic disease, there is no clearly defined systemic therapy. Metastasectomy is thought to improve progression-free survival, but in many cases like this, this strategy is not feasible.

In case reports hemangiopericytomas, entity closely related to solitary fibrous tumors, has achieved stable disease with the use of interferon with or without thalidomide. So far, this is the first case reported in the literature in which benefit is demonstrated with the use of interferon in a patient with a malignant variant renal solitary fibrous tumor with metastatic disease, achieving stable disease for about 20 months.

Some authors suggest the use of antiangiogenic therapies (bevacizumab, sunitinib, pazopanib, etc.), based on the findings of high vascularity and a possible origin of pericytes at this entity [15]. The combination of bevacizumab associated with temozolomide is a potentially promising scheme for patients with solitary fibrous tumors. A series of 14 patients with solitary fibrous tumor unresectable or metastatic, were treated with temozolomide 150 mg/m2 orally on days 1–7 and days 15–21 and bevacizumab 5 mg/kg intravenously on days 8 and 22, with cycles every 28 days. In this study, 11 patients (79%) achieved partial response assessed by Choi criteria with 2 cases (14%) with stable disease. The median progression-free survival was 8.6 months [12, 19, 20].

## Figures and Tables

**Table 1:** Summary of reported cases of renal SFT. (N.A. data not presented in the publication.)

Case	Reference	Age	Sex	Localization	Size (cms)	Subtype	Followup (months)	Outcomes
1	Fain et al. J Urol Pathol 1996; 4: 227–238	45	F	Right kidney	6	Benign	8	Tumor free
2	Fain et al. J Urol Pathol 1996; 4: 227–238	46	F	Right kidney	7,2	Benign	33	Tumor free
3	Fain et al. J Urol Pathol 1996; 4: 227–238	51	M	Left kidney	4,5	Benign	2	Tumor free
4	Gelb et al. Am J Surg Pathol 1996; 20: 1288–1295	48	F	Right kidney	3	Benign	1	Death from other cause Tumor free
5	Fukunaga and Nikaido Histopathology 1997; 30: 451–456	33	F	Right kidney	3,5	Benign	90	Tumor free
6	Fukunaga and Nikaido Histopathology 1997; 30: 451–456	36	F	Left kidney	2	Benign	12	Tumor free
7	Hasegawa et al. [2]	64	M	Kidney (laterality not reported)	4,5	Benign	8	Tumor free
8	Leroy et al. Urol Int 2000; 65: 49–52	66	F	Right kidney	9	Benign	9	Tumor free
9	Morimitsu et al. APMIS 2000; 108: 617–625	72	F	Right kidney	8	Benign	10	Tumor free
10	Yazaki et al. Int J Urol 2001; 8: 504–508	70	M	Right kidney	6	Benign	N.A.	N.A.
11	Wang et al. Am J Surg Pathol 2001; 25: 1194–1199	41	M	Right kidney	14	Benign	48	Tumor free
12	Wang et al. Am J Surg Pathol 2001; 25: 1194–1199	72	M	Right kidney	13	Benign	5	Tumor free
13	Cortes-Gutierrez et al. J Urol 2001; 166: 602	28	F	Left kidney	15	Benign	12	Tumor free
14	Magro et al. Pathol Res Pract 2002; 198: 37–43	31	F	Right kidney	8,6	Benign	8	Tumor free
15	Durand et al. Prog Urol 2003;13:491–494	35	M	Right kidney	17	Benign	6	Tumor free
16-17	Llarena Ibarguren et al. Arch Esp Urol 2003; 56: 835–840	51	F	Bilateral	25 (left) 2 (right)	Benign	N.A.	N.A.
18	Bugel et al. Prog Urol 2003; 13: 1397–1401	60	F	Right kidney	11	Benign	48	Tumor free
19	Gres et al. Prog Urol 2004; 14: 65–66	82	M	Right kidney	9	Benign	13	Tumor free
20	Yamada et al. Pathol Int 2004; 54: 914–917	59	M	Left kidney	6,8	Benign	N.A.	N.A.
21–27	Pierson et al. Mod Pathol 2005; 18: 159A	Median (52,6) range 29–79)	N.A.	N.A.	median (5,7), range 2,2–10)	Benign	N.A.	N.A.
28	Kawagoe et al. Nishinihon J Urol 2005; 67: 568–571	83	F	Left kidney	11	Benign	20	Tumor free
29	Johnson et al. J Comput Assist Tomogr 2005; 29: 481–483	51	F	Right kidney	11	Benign	N.A.	N.A.
30	Yamaguchi et al. Urology 2005; 65: 175	51	F	Left kidney	10	Benign	N.A.	N.A.
31	Kohl et al. Arch Pathol Lab Med 2006; 130: 117–119	85	F	Left kidney	3,5	Benign	N.A.	N.A.
32	Koroku et al. Hinyokika Kiyo 2006; 52: 705–706	18	F	Left kidney	3,2	Benign	15	Tumor free
33	Provance / Ferrari et al. Clin Pediatr (Phila) 2006; 45: 871–873	4	M	Right kidney	8	Benign	N.A.	N.A.
34	Fine et al. [3]	76	M	Left kidney	12	Malignant	4	Persistent tumor
35	Bozkurt et al. APMIS 2007; 115: 259–262	51	F	Left kidney	4	Benign	10	Tumor free
36	Znati et al. [10]	70	M	Left kidney	15	Benign	6	Tumor free
37	Constantinidis et al. Can J Urol 2007; 14: 3583–3587	26	M	Right kidney	5	Benign	6	Tumor free
38	Hirabayashi et al. Hinyokika Kiyo 2008; 54: 357–359	44	F	Left kidney	5,8	Benign	28	Tumor free
39	Magro et al. [11]	34	F	Left kidney	9	Malignant	15	Tumor free
40	Amano et al. Hinyokika Kiyo 2008; 54: 765–769	67	M	Left kidney	7	Benign	10	Tumor free
41	Yoneyama et al. Hinyokika Kiyo 2009; 55: 479–481	76	F	Right kidney	2,2	Benign	48	Tumor free
42	Hirano et al. [6]	75	M	Left kidney	4,5	Benign	9	Tumor free
43	Taxa et al. Actas Urol Esp 2010; 34: 568–570	39	F	Left kidney	2,5	Benign	12	Tumor free
44	Yamaguchi et al. Hinyokika Kiyo 2010; 56: 435–438	39	F	Left kidney	20	Benign	6	Tumor free
45	Marzi et al. Minerva Urol Nefrol 2011; 63: 109–113	72	F	Left kidney	19	Malignant	N.A.	N.A.
46	Hsieh et al. [8]	50	F	Right kidney	9	Malignant	30	Tumor free
47	De Martino et al. [5]	68	F	Left kidney	7	Malignant	5	Death by the disease
48	Caso actual	49	F	Left kidney	9,8	Malignant	23	Stable disease

**Table 2:** Clinicopathologic features and outcomes of the 49 cases reported with solitary fibrous tumors of the kidney.

Median age in years (range)	51 (4–85)
Sex	
Male	14
Female	28
Unknown	7
Location	
Left kidney	23
Right kidney	17
Bilateral	1
Unknown	8
Site	
Kidney	33
Renal capsule	6
Peripelvis	3
Pelvis	1
Unknown	6
Medium size in cm (range)	7,6 (2–20)
Histology	
Benign	42
Malignant	7
Treatment	
Tumor resection	2
Nephrectomy	41
Unknown	6
Subcutaneous interferon	1
Outcome	
No evidence of disease	25
Metastasis	4
Unknown	20
